# Ultraviolet germicidal irradiation of the inner bore of a CT gantry

**DOI:** 10.1002/acm2.13067

**Published:** 2020-11-18

**Authors:** Mahadevappa Mahesh, Jeffrey H. Siewerdsen

**Affiliations:** ^1^ The Russell H. Morgan Department of Radiology Johns Hopkins University Baltimore MD USA; ^2^ Department of Biomedical Engineering Johns Hopkins University Baltimore MD USA

**Keywords:** coronavirus, COVID‐19, germicidal irradiation, hygiene, medical imaging, ultraviolet, viral decontamination

## Abstract

**Purpose:**

To investigate the feasibility and practicality of ultraviolet (UV) germicidal irradiation of the inner bore of a computed tomography (CT) gantry as a means of viral decontamination.

**Method:**

A UV lamp (PADNUT 38 W, 253 nm UV‐C light tube) and UV‐C dosimeter (GENERAL UV‐C Digital Light Meter No. UV512C) were used to measure irradiance throughout the inner bore of a CT scanner gantry. Irradiance (units μW/cm^2^) was related to the time required to achieve 6‐log viral kill (10^−6^ survival fraction).

**Results:**

A warm‐up time of ~120 s was required for the lamp to reach stable irradiance. Irradiance at the scan plane (z = 0 cm) of the CT scanner was 580.9 μW/cm^2^, reducing to ~350 μW/cm^2^ at z = ±20 cm toward the front or back of the gantry. The angular distribution of irradiation was uniform within 10% coefficient of variation. A conservative estimate suggests at least 6‐log kill (survival fraction ≤ 10^−6^) of viral RNA within ±20 cm of the scan plane with an irradiation time of 120 s from cold start. More conservatively, running the lamp for 180 s (3 min) or 300 s (5 min) from cold start is estimated to yield survival fraction <<10^−7^ survival fraction within ±20 cm of the scan plane.

**Conclusion:**

Ultraviolet irradiation of the inner bore of the CT gantry can be achieved with a simple UV‐C lamp attached to the CT couch. Such practice could augment manual wipe‐down procedures, improve safety for CT technologists or housekeeping staff, and could potentially reduce turnover time between scanning sessions.

## 1 INTRODUCTION

Medical imaging — and computed tomography (CT) in particular — is a vital component of the medical diagnostic arsenal. The utilization of imaging resources can be greatly compromised in periods of contagious viral pandemic, requiring time‐consuming decontamination between scan sessions to minimize the risk of infection between patients on the same scanner. For example, manual wipe‐down of a CT scanner suite plus negative airflow changeover in the scanner room between sessions is estimated to require 45–60 min. Manual wipe‐down (particularly in difficult to reach areas with relatively high exposure to exhalants from patients, such as the inner bore of the scanner) presents potentially increased viral exposure to CT scanner technologists and/or cleaning staff.

Ultraviolet (UV) light (viz., 254 nm ultraviolet‐C, UV‐C) has demonstrated utility in germicidal irradiation of surfaces in healthcare settings.^1^, ^2^, ^3^, ^4^ It has also been demonstrated of value in decontamination of personal protective equipment (PPE) in relation to coronavirus and other viral respiratory syndromes.^5^, ^6^, ^7^, ^8^ We report the potential utility of ultraviolet (UV‐C) germicidal irradiation to augment the cleaning of CT scanners, specifically addressing the task of disinfecting the inner bore of the CT gantry using a UV lamp suspended from the head of the CT couch. Since cleaning the inner bore is an important but cumbersome, non‐ergonomic aspect of disinfecting the scanner, such capability could ease the burden on the CT technologists or the cleaning staff and help to reduce turnover time.

## 2 **MATERIALS AND METHOD**S

**Fig. 1:**
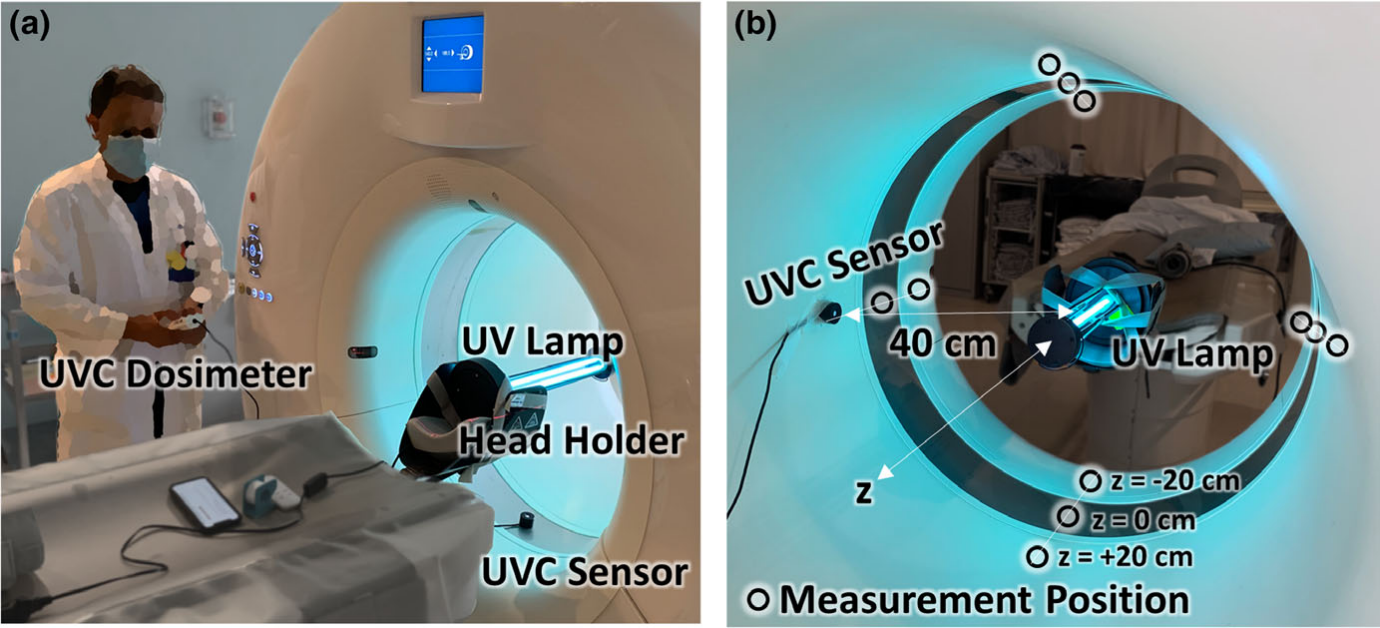
Experimental setup for UV‐C dose measurement in a computed tomography scanner gantry. (a) Arrangement of the lamp (suspended from a head holder) and sensor within the gantry. The operator controls the lamp via remote control and reads the dosimeter safely from outside line‐of‐sight to the lamp. (b) Illustration of 12 sensor measurement positions within the bore — at 4 cardinal locations at *z* = −20, 0, and + 20 cm from the central scan plane.

The ultraviolet lamp used in this work was a PADNUT UV‐C cleaning light tube for air purification and room/surface cleaning. The lamp has a power of 38 W with a pair of 36 cm length light tubes specified to emit 253 nm UV‐C light. As shown in Fig. 1(a), the lamp was suspended horizontally by securing its base in a carbon fiber head holder with Velcro straps at the isocenter of a Definition Flash CT scanner (Siemens Healthineers, Forchheim Germany). The long axis of the lamp was aligned with the longitudinal (*z*) axis of the gantry, and the midpoint of the lamp was placed at the scan plane (*z* = 0 cm). The lamp was operated via a remote control, allowing the operators to stand clear of the area when the lamp was on.

The UV‐C dosimeter used in this work was a GENERAL UV‐C Digital Light Meter (No. UV512C) with a specified sensitivity range of 220 to 275 nm and calibration point 254 nm corresponding to the wavelength of UV‐C. The dosimeter was operated in the “low” illumination range (specified as 1–9999 μW/cm^2^) with specified accuracy at room temperature of ±4%.

The sensor was placed on the inner bore of the CT scanner gantry at 4 cardinal positions as shown in Fig. 1(b): at the scan plane (denoted *z* = 0 cm); at *z* = −20 cm toward the “front” of the gantry (i.e., toward the CT table); and at *z* = +20 cm toward the “back” of the gantry. The inner gantry diameter was 80 cm, so the nominal distance from the lamp to the sensor was 40 cm.

The warm‐up time of the lamp was first assessed by measuring the irradiance (units of micro‐Watt per centimeter squared, μW/cm^2^) as a function of time repeatedly from cold start. Then, the irradiance was measured at each of the sensor positions noted above, recording measurements at 30 s intervals up to 180 s.

As reported by Tseng and Li,^9^ the dose (i.e., fluence, φ, with units milli‐Joules per centimeter squared, mJ/cm^2^) required to achieve 6‐log viral kill (10^−6^ survival fraction) is 30 mJ/cm^2^. This dose target value represents a nominal value above which survival fraction is expected to be <10^−6^ for viruses with single‐stranded RNA. The measured irradiance ($E_{e}$) was related to the time (t) required for 6‐log viral kill as ($\varphi /E_{e}$), noted with explicit units as follows:

$$t\left(s\right)=\frac{\varphi }{E_{e}}=\left[\varphi \left(\frac{\text{mJ}}{{\text{cm}}^{2}}\right)\right]\left[\frac{1}{E_{e}}\left(\frac{{\text{cm}}^{2}}{\mu W}\right)\right]\left[1000\left(\mu W\frac{s}{\mu J}\frac{\mu J}{\text{mJ}}\right)\right]$$

For example, an irradiance of $E_{e}$ = 500 μW/cm^2^ provides 6‐log kill in t = (30 × 1000/500) = 60 s (1 min).

## 3 RESULTS

**Fig. 2:**
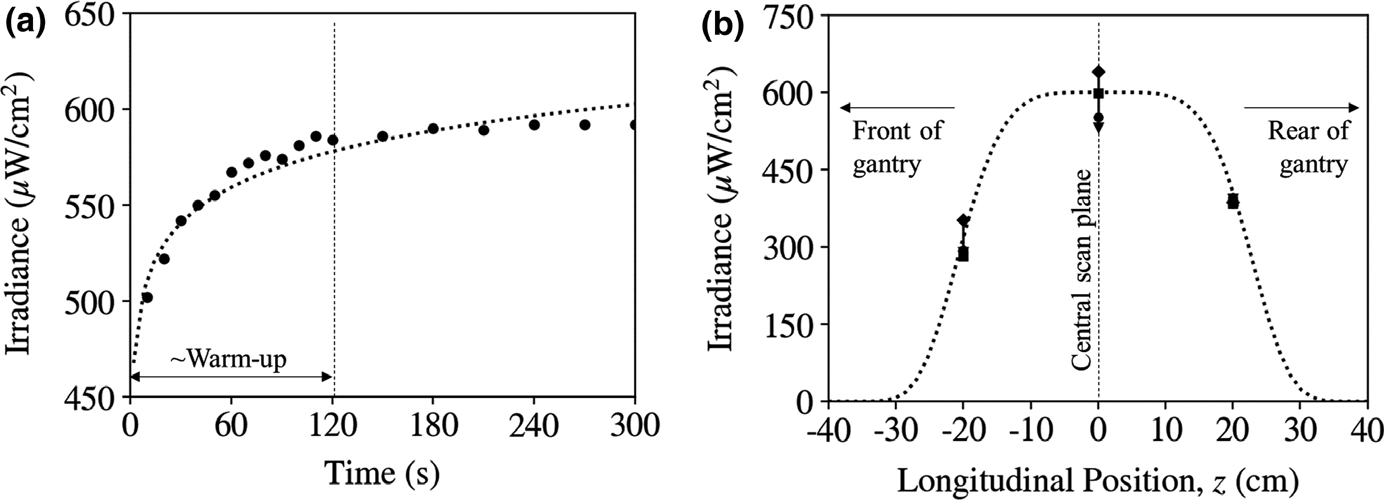
Irradiance measured for the UV‐C lamp at a distance of 40 cm. (a) Irradiance measured versus time from switching the lamp on, suggesting a warm‐up time of ~ 120 s. (b) Irradiance measured as a function of z position within the gantry, with negative or positive z denoting the front or back of the gantry, respectively. The error bars and multiple plot symbols at a particular *z* position correspond to measurements at four cardinal locations within the gantry.

The lamp exhibited a considerable warm‐up time as exhibited in Fig. 2(a), requiring at least 120 s to reach constant irradiance. Results reported below were averaged over measurements recorded at 120, 150, and 180 s from start.

The mean irradiance at the *z* = 0 scan plane was (580.9 ± 48.9) μW/cm^2^. The irradiance decreased measurably along the *z* direction as shown in Fig. 2(b): (305.0 ± 32.4) μW/cm^2^ at *z* = −20 cm toward the front of the gantry; and (390.3 ± 5.6) μW/cm^2^ at *z* = +20 cm toward the back of the gantry. The slightly steeper decline at negative *z* (toward the front of the gantry) was likely due to shadowing from the base of the lamp and/or head holder.

The coefficient of variation in irradiance over the four cardinal positions measured was within ~10% at all *z* positions — for example, (48.9/580.9 μW/cm^2^) = 8.4% at the *z* = 0 scan plane. The small angular dependence was likely due to slight mis‐centering of the lamp within the gantry and perhaps to variations from the lamp itself or due to shadowing of the three small support rods in the lamp assembly. The variation in irradiance with angle is evident in the error bars of Fig. 2(b).

We relate the measured irradiance to an estimate of the time required for 6‐log kill (φ = 30 mJ/cm^2^), bearing in mind numerous practical considerations. An overly optimistic estimate is obtained at the *z* = 0 scan plane, where the measured irradiance of 580.9 μW/cm^2^ suggests 6‐log kill in t = 52 s (less than a minute). Taking instead the minimum irradiance at *z* = ±20 cm as the more pertinent measurement (305.0 μW/cm^2^), the time required is t = 98 s.

Neither of these estimates account for the warm‐up time of the lamp (~120 s) during which time the irradiance varies by nearly a factor of 2 from cold start as shown in Fig. 2(a). Integrating over the warm‐up curve [logarithmic fit of Fig. 2(a): $E_{e}=\left(E_{\max}/2\right)+26.9*\ln\left(t\right)$] and again taking the estimate of the minimum irradiance at ±20 cm, 6‐log kill is achieved in t~ 120 s from cold start.

A conservative, practical procedure could instead simply run the lamp for 180 s from cold start, in which time the fluence delivered to the *z* = 0 cm and ±20 cm locations (including integration along the warm‐up curve) is 102 and 48 mJ/cm^2^, respectively. Alternatively, and considering a hypothetical turnover time of 10 min (including roughly 5 min for setup and 5 min irradiation), running the lamp for 300 s (5 min) from cold start delivers 174 and 84 mJ/cm^2^ at the *z* = 0 cm and ±20 cm locations, respectively. From the data reported by Tseng and Li,^9^ these fluence levels correspond to <<10^−7^ survival fraction (>99.99999% viral kill).

Measurements were not performed beyond *z* = ±20 cm, and the simple extrapolation (flat‐top Gaussian fit) in Fig. 2(b) suggests that irradiance is minimal at *z* = ±30 cm, where the bore of the gantry also tends to be curved, further diminishing the absorbed fluence by the cosine of the incident angle.

## 4 DISCUSSION AND CONCLUSIONS

This work reports the basic feasibility and practicality of UV germicidal irradiation of the inner bore of a CT scanner in terms of the irradiance and time required to achieve at least 6‐log viral kill. A simple setup was realized in which a UV‐C lamp was suspended within the gantry bore attached to the head of the CT couch. Total cost of materials at the time of this study was $105 for the lamp plus $416 for the dosimeter. The spatial distribution of irradiance within the bore was measured, and the results imply at least 6‐log kill in ~120 s (2 min) of irradiation.

The reported measurements were performed on a Siemens Definition Flash CT scanner, giving a distance of 40 cm from the lamp (placed at isocenter) to the sensor (placed on the inner surface of the CT gantry bore). For CT scanners with a similar gantry bore size (80 cm), the measurements reported above should apply directly. The irradiance as a function of distance was not measured in the current work. Based on simple consideration of Gauss’ law for point‐ and line‐sources, the reduction in irradiance with distance is likely between $\left(1/r^{2}\right)$ and (1/r), respectively. Taking the former as a conservative estimate for systems with a different bore diameter, the measurements reported above could be approximately scaled to a different radius (R, in cm) by the factor ${\left(40/R\right)}^{2}$.

A variety of limitations of the current study deserve acknowledgment. It is worth noting that the output of the UV lamp may diminish over time, and the usable lifespan of the lamp was not investigated in the current work. Judicious quality assurance could involve periodic constancy checks or measurement of irradiance at the time of decontamination to ensure proper dose delivered during decontamination. Moreover UV light is known to discolor certain plastics and could affect material integrity of the gantry covers. Such effects were not studied in the current work. The current study was limited to a single model of UV lamp, and many other lamps are available. The current model was selected simply due to its availability at the time of the study. Specifications that would improve performance include increased power, reduced warm‐up time, and/or longer length. Finally, it is important to note that contaminants could reside in crevices in the scanner cover that may be shadowed from UV‐C irradiation.

This work should not be interpreted as recommending UV‐C irradiation as replacement to manual wipe‐down with disinfectant; rather, the setup reported here could augment such procedures. Because manual wipe‐down cleaning within the bore of the gantry may be difficult and non‐ergonomic, it is prone to incomplete manual disinfection. Augmenting such procedures with UV‐C irradiation could ensure more thorough and consistent decontamination while easing the burden on the CT technologists or the cleaning staff. Because the results suggest sufficient levels of germicidal dose within minutes, the procedure could potentially reduce the turnover time between scan sessions. The full workflow of manual wipe‐down plus UV‐C decontamination is the subject of future work. Given the ease of use and fairly rapid irradiation times, one may envision an increasing role of UV‐C germicidal irradiation within medical imaging hygiene protocols in the future, or even scanners with capability for germicidal irradiation built into the scanner. Similarly, MR scanners present an even more challenging wipe‐down procedure to the technologists or cleaning staff and use of UV‐C germicidal irradiation with MR compatible UV‐C lamps is being considered for future work.
